# Protective Effect of Shexiang Baoxin Pill on Myocardial Ischemia/Reperfusion Injury in Patients With STEMI

**DOI:** 10.3389/fphar.2021.721011

**Published:** 2021-09-10

**Authors:** Haixia Qin, Siyuan Li, Zhenbing Liu

**Affiliations:** Ordos Central Hospital, Ordos Clinical Medical College, Inner Mongolia Medical University, Ordos, China

**Keywords:** shexiang baoxin pill, ST-segment elevation myocardial infarction, myocardial ischemia reperfusion injury, myocardial protection, myocardial salvage

## Abstract

**Background:** There is no definite effect in the treatment of myocardial ischemia/reperfusion (I/R) injury in patients with acute ST-segment elevation myocardial infarction (STEMI). We evaluated the protective effect of Shexiang Baoxin Pill (SBP) on I/R injury in STEMI patients.

**Methods:** STEMI patients were randomly divided into a primary percutaneous coronary intervention (PPCI) group (n = 52) and a PPCI + SBP group (n = 51). The area at risk of infarction (AAR) and final infarct size (FIS) were examined by single-photon emission computed tomography (SPECT). I/R injury was assessed using myocardial salvage (MS) and salvage index (SI) calculated from AAR and FIS.

**Results:** The ST-segment resolution (STR) in the PPCI + SBP group was significantly higher than that in the PPCI group (*p* = 0.036), and the peak value of high-sensitivity troponin T (hsTNT) was lower than that in the PPCI group (*p* = 0.048). FIS in the PPCI + SBP group was smaller than that in the PPCI group (*p* = 0.047). MS (*p* = 0.023) and SI (*p* = 0.006) in the PPCI + SBP group were larger than those in the PPCI group. The left ventricular ejection fraction (LVEF) in the PPCI + SBP group was higher than that in the PPCI group (*p* = 0.049), and N-terminal pro-B type natriuretic peptide (NT-proBNP) level in the PPCI + SBP group was lower than that in the PPCI group (*p* = 0.048).

**Conclusions:** SBP can alleviate I/R injury (MS and SI), decrease myocardial infarction area (peak value of hsTNT and FIS), and improve myocardial reperfusion (MBG and STR) and cardiac function (LVEF and NT-proBNP).

## Introduction

ST-segment elevation myocardial infarction (STEMI) is the main cause of death in patients with coronary heart disease (CHD), and its incidence is increasing (GBD 2015 Disease and Injury Incidence and Prevalence Collaborators, 2018). With the continuous progress of various treatment measures including reperfusion treatment, the mortality of STEMI patients has been significantly reduced (Bulluck et al., 2016). However, reperfusion treatment can induce myocardial ischemia/reperfusion (I/R) injury Hausenloy and Yellon (2015), which can result in poor prognosis (Bolognese et al., 2004). Therefore, effective treatment of I/R injury is critical. At present, no measures have been found for effective treatment of I/R injury in STEMI patients (Hausenloy et al., 2017). The pathogenesis of I/R injury is diverse Hausenloy et al. (2017), and cooperative treatment through different mechanisms may be a new approach for I/R injury treatment (Davidson et al., 2019). Shexiang Baoxin Pill (SBP) is a traditional Chinese medicine compound preparation, it is mainly composed of artificial moschus, radix ginseng, calculus bovis artifactus, cortex cinnamomi, styrax, venenum bufonis, and borneolum syntheticum, and is mainly used to treat CHD (Lu et al., 2018; Ge et al., 2021). The extract of the components of SBP reduces I/R injury, myocardial remodeling, and myocardial infarction area in animals through multiple mechanisms. These multiple mechanisms of action may provide a basis for the use of SBP in the treatment of STEMI (Wu et al., 2011; Fancelli et al., 2014; Zheng et al., 2017; Du et al., 2018a; Du et al., 2018b; Li et al., 2018; Li et al., 2020). Recent animal studies have found that SBP can reduce I/R injury and final infarct size (FIS) (Xiang et al., 2013). Small clinical trials with low methodological quality and ill-defined bias risk have demonstrated that SBP decreased the level of myocardial injury markers and main adverse cardiovascular events (MACCE) in patients with acute myocardial infarction (AMI) (Lu et al., 2018). SBP added to conventional treatment may have beneficial effects on the long-term outcomes of Non-ST-elevation acute coronary syndromes (NSTE-ACS) (Zhou et al., 2016). Therefore, SBP may reduce myocardial I/R injury and myocardial infarction area in human patients. Thus, the current study was designed to examine the therapeutic effect of SBP in I/R injury of STEMI patients.

## Materials and Methods

### General Information

**FIGURE 1:**
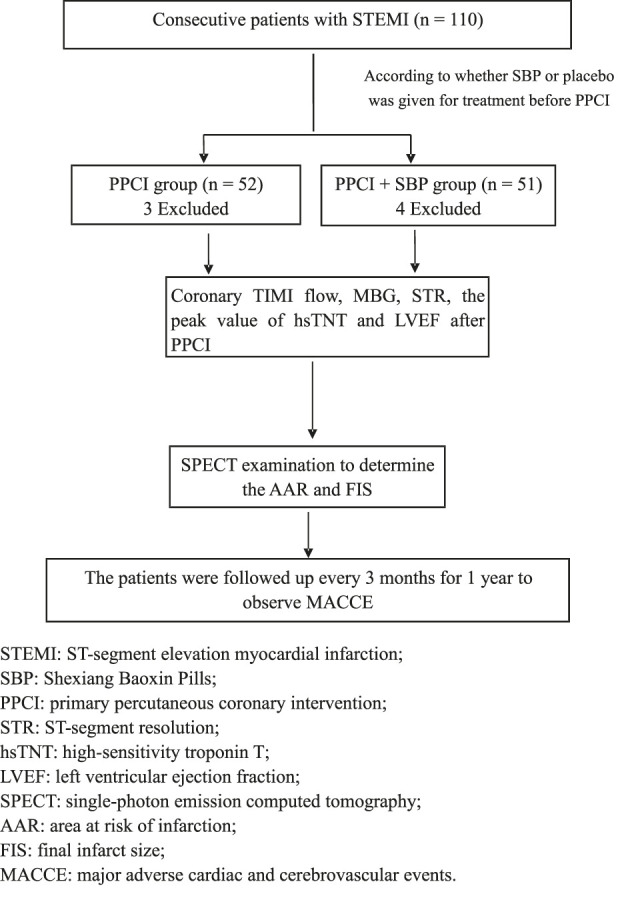
Technical route.

From September 1, 2016 to March 31, 2019, 110 patients with STEMI in the Second District of Cardiology Department of Ordos Central Hospital were selected to receive primary percutaneous coronary intervention (PPCI). The inclusion criteria included: age ≥18 years; chest pain <12 h; and the first electrocardiogram (ECG) showed ST segment elevation ≥0.1 mV in two or more adjacent leads. Exclusion criteria included the following: advanced malignant tumor; history of myocardial infarction; acute inflammatory or infectious disease; decompensated liver disease; end-stage renal disease; severe heart failure (Killip III-IV); history of coronary artery bypass; hypersensitivity to SBP; failure to complete the study; and lack of follow-up. The selected patients were randomly divided into the PPCI group (52 cases, two patients had a history of myocardial infarction and one had severe heart failure (Killip III). A total of three patients were excluded from the standard) and the PPCI + SBP group (51 cases, 1 case had a history of myocardial infarction, 2 cases had severe heart failure (Killip III and Killip IV), 1 case had acute inflammation. A total of 4 cases were excluded) according to whether they were treated with SBP or not. All patients were treated with conventional medicine and PPCI implemented as early as possible 12 h from the onset of the symptoms according to the guidelines. SBP and placebo were given immediately after routine medication. The PPCI + SBP group was given two pills of SBP (Shanghai Hutchison Pharmaceuticals, 22.5 mg/pill) immediately after admission as soon as possible, and then two pills each time, three times a day, during 1 month. The PPCI group was given placebo in the same way (Figure 1). The general baseline characteristics of all patients, including gender, age, smoking history, previous hypertension, diabetes, cerebral infarction history, coronary heart disease family history, admission heart rate, systolic blood pressure, diastolic blood pressure, creatinine, blood glucose, low-density lipoprotein, high-sensitivity troponin T (hsTNT), and myocardial ischemia time, were recorded.

The research plan was approved by the ethics committee of Inner Mongolia Medical University. All patients provided written informed consent.

### Preoperative, Intraoperative, and Postoperative Medication

All patients were given aspirin tablets (300 mg), clopidogrel tablets (<75 years old, 600 mg; or ≥75 years old, 300 mg) or ticagrelor 180 mg (<75 years old) before PPCI. Intravenous heparin (100 IU /kg) was used. If the patient’s thrombus load was heavy (thrombus grade ≥3), tirofiban (10 μg/kg) was given, then intravenous tirofiban (0.15 μg/kg/min) was maintained for 24 h after which heparin was changed to 50–70 IU/kg. Other medications included aspirin 75–100 mg (once a day) for life, clopidogrel 75 mg (once a day) or ticagrelor 90 mg (twice a day) (less than 75 years old) for at least 1 year. Other drugs, including angiotensin-converting enzyme inhibitor (ACEI), angiotensin receptor blockers (ARB), β-blockers, and statins, were used according to published guidelines.

### Experimental Instruments and Methods

#### Biological Index Detection

HsTNT was measured in peripheral venous blood of all patients at admission and every 6 h after admission until the peak of hsTNT appeared. HsTNT was determined by Elecsys 2010, Roche, Switzerland. Peripheral venous blood was drawn 24 h after admission, N-terminal pro-B type natriuretic peptide (NT-proBNP) was determined by Elecsys 2010, Roche, Switzerland. Blood routine examination, liver and kidney function, blood lipid and blood glucose, blood potassium, sodium and chlorine were measured conventionally.

#### Process of Coronary Angiography and PPCI

PPCI was performed through radial artery or femoral artery with a routine 6 F catheter, guide wire and balloon dilator. According to the thrombus load of the target vessel (thrombus grade ≥3) Gibson et al. (2001), aspiration thrombectomy was used. Thrombolysis in myocardial infarction (TIMI) and myocardial blush grade (MBG) van 't Hof et al. (1998) were used to evaluate the coronary flow before and after operation. TIMI was divided into 0–3 grades according to the filling condition of coronary artery contrast medium. Grade 0 indicated total occlusion without blood flow passing through, while grade 3 marked normal blood flow passing through the coronary artery (TheStudy Group (198, 1985). MBG was divided into 0–3 grades according to the myocardial development, with grade 0 indicating no myocardial development, and grade 3 denoting normal myocardial development (Gibson et al., 2001). Angiographic assessment was performed offline by two experienced observers blinded to the PPCI + SBP group or PPCI group, electrocardiographic and echocardiographic data, and the single-photon emission computed tomography (SPECT) imaging results. Disagreements between the two observers were resolved by joint evaluation of the data and discussion.

#### ECG Examination

ECG was reexamined 90 min after PPCI. The ST segment evaluation of ECG was also evaluated by two independent experienced professional doctors without knowledge of patient group assignment. The ST-segment resolution (STR) was recorded.

#### Single-Photon Emission Computed Tomography

All patients underwent SPECT (e.cam, Siemens) to evaluate the area at risk of infarction (AAR) 24 h to 7 days after PPCI. ^99^Technetium-sestamibi (^99^Tcm-MIBI, Beijing Senke company or 401 atomic High Tech) 700 MBq was injected intravenously into the patients first, and then detected with the double probe rotating gamma camera-automatic direct analog computer (ADAC) with a high resolution flat hole collimator within 8 h after injection. The final infarct size (FIS) was evaluated by SPECT 30 days after PPCI (the patients rested in supine position for 30 min, and then were detected by ADAC after intravenous injection of ^99^Tc^m^-MIBI 700 MBq for 60 min). AAR and FIS were mainly lower than normal pixels on the myocardium (AAR and FIS are expressed by the percentage of measured area in the left ventricular area and were calculated by quantitative perfusion software (QPS)). Two fully blinded operators, using an off line dedicated workstation, performed all measurements. Myocardial salvage (MS) was defined as the difference between infarct risk myocardium and infarct myocardial area (AAR−FIS). The salvage index (SI) was calculated as MS/AAR.

#### Echocardiography

All the selected patients were examined by two fully blinded operators using echocardiography (Vivid 7, GE) within 1 week, and left ventricular ejection fraction (LVEF) was measured using the Simpson method.

## Clinical End Point Events

All patients were followed up by telephone or clinic every 3 months for at least 1 year. The main clinical end points were MACCE, including cardiac death, non-fatal myocardial infarction, new heart failure, new cerebral infarction, and transient cerebral ischemia attack (all the end points were confirmed by hospital medical records or contacting a competent doctor). Non-fatal reinfarction and new heart failure were diagnosed according to the existing diagnostic criteria. New cerebral infarction and transient ischemic attack were diagnosed by brain CT or MRI, and a neurologist was invited for diagnosis. MACCE records the occurrence according to the sequence of cardiac death > non-fatal myocardial infarction > new heart failure > new cerebral infarction > transient cerebral ischemia attack, so as to avoid repeated recording of cardiovascular and cerebrovascular events.

## Statistical Analysis

SPSS17.0 (SPSS Inc., Chicago, Illinois, United States) was used for data analysis, and differences were considered statistically significant at *p* < 0.05. The measurement data is expressed as mean ± standard deviation (x ± s). Independent *t* test was used to evaluate the difference between the PPCI group and PPCI + SBP group. Variables of a non-normal distribution (determined by Kolmogorov-Smirnov test) are expressed as median and quartile ranges and were compared using Wilcoxon rank sum test. The counting data are expressed as the percentage composition ratio (%) and were compared using the Chi-square test or Fisher’s exact test. The Kaplan-Meier method was used to analyze MACCE cumulative events graphically.

## Results

### Comparison of Baseline and Clinical Characteristics

Seven of the 110 STEMI patients were excluded. There was no significant difference in baseline characteristics between the two groups (Table 1). There was no significant difference in preoperative, intraoperative, and postoperative medication between the two groups (*p* > 0.05) (Table 2).

**TABLE 1:** Comparison of clinical characteristics between PPCI + SBP and PPCI groups.

—	PPCI Group (n = 52)	PPCI + SBP Group (n = 51)	P
Age, years	58.6 ± 11.3	57.2 ± 9.6	0.513
Gender (male), n (%)	42 (80.8)	43 (84.3)	0.636
History of Smoking, n (%)	34 (65.4)	34 (66.7)	0.891
History of hypertension, n (%)	23 (44.2)	22 (43.1)	0.769
History of diabetes, n (%)	8 (15.4)	7 (13.7)	0.811
History of cerebral infarction, n (%)	3 (5.8)	2 (3.9)	0.663
Admission Heart Rate, bpm	70.5 ± 14.1	72.5 ± 12.6	0.451
Systolic blood pressure, mmHg	126.4 ± 24.7	125.0 ± 24.9	0.778
Diastolic blood pressure, mmHg	81.7 ± 17.3	79.8 ± 15.4	0.560
creatinine, umol/L	78.3 ± 19.5	76.3 ± 15.7	0.555
Blood glucose, mmol/L	7.9 ± 3.2	7.7 ± 3.8	0.781
Low density lipoprotein, mmol/L	3.2 ± 1.4	3.2 ± 0.8	0.991
hsTNT at admission, ug/L	1.10 ± 2.17	0.72 ± 1.56	0.314
The Peak Value of hsTNT, ug/L	7.09 ± 4.51	5.54 ± 3.23	0.048
NT-proBNP after admission, pg/ml	1,014.86 ± 1,259.38	567.02 ± 988.82	0.048
ST-segment resolution, %	63.30 ± 22.14	73.04 ± 24.25	0.036
LVEF, %	53.27 ± 9.09	56.53 ± 7.42	0.049
Myocardial ischemia time, h	7.53 ± 9.18	6.21 ± 2.93	0.331

HsTNT, high-sensitivity troponin T; LVEF, Left ventricular ejection fraction.

**TABLE 2:** Comparison of preoperative, intraoperative and postoperative medication between PPCI + SBP and PPCI.

—	PPCI Group (n = 52)	PPCI + SBP Group (n = 51)	P
heparin, n (%)	52 (100.0)	51 (100.0)	1.000
aspirin, n (%)	52 (100.0)	51 (100.0)	1.000
clopidogrel, n (%)	27 (51.9)	19 (37.3)	0.134
ticagrelor, n (%)	26 (50.0)	32 (62.7)	0.192
tirofiban, n (%)	20 (38.5)	18 (35.3)	0.739
ACEI, n (%)	42 (80.8)	40 (78.4)	0.768
ARB, n (%)	5 (9.6)	5 (9.8)	0.974
β-blocker, n (%)	46 (88.5)	46 (90.2)	0.776
statins, n (%)	52 (100.0)	51 (100.0)	1.000

ACEI: angiotensin-converting enzyme inhibitor; ARB: angiotensin receptor blockers.

### Intraoperative and Postoperative Conditions

#### Intraoperative Conditions

There was no significant difference in TIMI preoperative blood flow classification (*p* = 0.649), TIMI postoperative blood flow classification (*p* = 0.438), Number of diseased blood vessel (*p* = 0.417), target blood vessel (*p* = 0.603), thrombus aspiration (*p* = 0.261), stent implantation (*p* = 0.414), and average number of stents (*p* = 0.81) between the two groups, while the MBG postoperative grade of the PPCI + SBP group was better than that of the PPCI group (*p* = 0.005) (Table 3).

**TABLE 3:** Comparison of intraoperative characteristics between PPCI + SBP and PPCI groups.

—	PPCI Group (n = 52)	PPCI + SBP roup (n = 51)	P
TIMI preoperative, n (%)	—	—	0.649
0	28 (53.8)	22 (43.1)	—
1	7 (13.5)	11 (21.6)	—
2	10 (19.2)	11 (21.6)	—
3	7 (13.5)	7 (13.7)	—
TIMI postoperative, n (%)	—	—	0.438
0	0 (0.0)	0 (0.0)	—
1	2 (3.8)	1 (2.0)	—
2	9 (17.3)	5 (9.8)	—
3	41 (78.8)	45 (88.2)	—
MBG postoperative, n (%)	—	—	0.005
0	0 (0.0)	0 (0.0)	—
1	4 (7.7)	2 (3.9)	—
2	30 (57.7)	15 (29.4)	—
3	18 (34.6)	34 (66.7)	—
Number of diseased vessels, n (%)	—	—	0.417
1	11 (21.2)	14 (27.5)	—
2	16 (30.8)	19 (37.3)	—
3	25 (48.1)	18 (35.3)	—
Target Vessel, n (%)	—	—	0.603
Left anterior descending artery	23 (44.2)	27 (52.9)	—
circumflex artery	5 (9.6)	3 (5.9)	—
right coronary artery	24 (46.2)	21 (41.2)	—
aspiration thrombectomy, n (%)	14 (26.9)	19 (37.3)	0.261
Average number of stents, n (%)	1.33 ± 0.73	1.29 ± 0.61	0.81
stent implantation, n (%)	48 (92.3)	49 (96.1)	0.414

TIMI, thrombolysis in myocardial infarction; MBG, myocardial blush grade.

#### Comparison of Postoperative Myocardial Reperfusion Assessed by STR and MBG

There was no significant difference in TIMI blood flow after PPCI between the PPCI + SBP group and PPCI group (*p* = 0.438) (Table 3). However, the MBG grade of the PPCI + SBP group was better than that of the PPCI group after PPCI (*p* = 0.005) (Table 3). In addition, the STR in the PPCI + SBP group was significantly higher than that in the PPCI group (63.30 ± 22.14% vs 73.04 ± 24.25%, respectively, *p* = 0.036) (Table 1). These results confirm that SBP ameliorate myocardial reperfusion.

### I/R Injury Comparison Evaluated by SPECT

There was no significant difference between the two groups regarding the AAR [8.00% (3.00–25.75) vs 18.00% (5.25–29.00), respectively, *p* = 0.234], but the MS [2.50% (0.125–9.750) vs 1.00% (−1.30–3.50), respectively, *p* = 0.023] and the SI [27.95% (0.00–45.00) vs 4.30% (−18.35–20.55), respectively, *p* = 0.006] were significantly improved in the PPCI + SBP group as compared to the PPCI group (Table 4). These indicate that SBP can alleviate I/R injury and rescue ischemic myocardium.

**TABLE 4:** Myocardial I/R injury and Final infarct size assessed by SPECT.

—	PPCI group (n = 52)	PPCI + SBP group (n = 51)	P
Area at risk of infarction (AAR), LV%	18.00 (5.25–29.00)	8.00 (3.00–25.75)	0.234
	(n = 43)	(n = 41)	
Final Infarct Size (FIS), LV%	15.00 (4.00–29.50)	6.50 (3.00–15.00)	0.047
	(n = 52)	(n = 51)	
Myocardial salvage (MS), LV%	1.00 (−1.30–3.50)	2.50 (0.125–9.750)	0.023
	(n = 29)	(n = 28)	
Salvage index (SI), %	4.30 (−18.35–20.55)	27.95 (0.00–45.00)	0.006
	(n = 29)	(n = 28)	

LV%, % of the left ventricle; SPECT, single-photon emission computed tomography; I/R, ischemia reperfusion; MS = AAR-FIS; SI = MS/AAR.

### Comparison of Myocardial Infarction Area by SPECT Evaluation and hsTNT

Compared with the PPCI group, the peak value of hsTNT in the PPCI + SBP group decreased (5.54 ± 3.23 ug/L vs 7.09 ± 4.51 ug/L, respectively, *p* = 0.048) (Table 1). SPECT showed that the FIS of the PPCI + SBP group decreased [6.50% (3.00–15.00) vs 15.00% (4.00–29.50), respectively, *p* = 0.047] as compared to PPCI group (Table 4), which mean that SPB can lower myocardial infarction area.

### Comparison of Cardiac Function Evaluated by LVEF and NT-proBNP

Compared with the PPCI group, the PPCI + SBP group had higher LVEF (56.53 ± 7.42% vs 53.27 ± 9.09%, respectively, *p* = 0.049), and lower NT-proBNP (567.02 ± 988.82 pg/ml vs 1,014.86 ± 1,259.38 pg/ml, respectively, *p* = 0.048) (Table 1). These results show that SBP improve cardiac function.

### Clinical End Point Events

**FIGURE 2:**
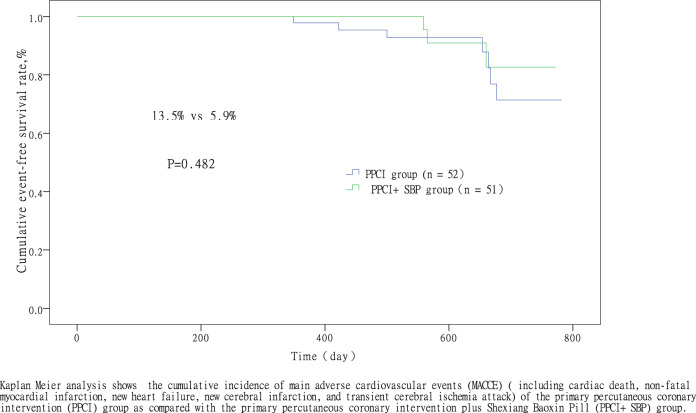
Comparison of the cumulative incidence of MACCE between the PPCI and PPCI + SBP group.

All patients were followed through January 31, 2019. There was no significant difference between the two groups (556.9 ± 191.5 days vs 483.1 ± 205.0 days, respectively, *p* = 0.062). MACCE occurred in 10 (9.7%) of all eligible patients: 7 cases (13.5%) in the PPCI group and 3 cases (5.9%) in the PPCI + SBP group. In the PPCI group, MACCE included 1 (1.9%) cardiac death, 3 (5.8%) non-fatal reinfarction, and 3 (5.8%) new heart failure, while in the PPCI + SBP group, MACCE included 2 (3.9%) non-fatal reinfarction and 1 (2.0%) case of new heart failure. There was no significant difference in the incidence of MACCE (13.5 vs 5.9%, *p* = 0.482) between the PPCI group and the PPCI + SBP group (Figure 2), which mean decreased MACCE trend, but there was no statistical difference.

## Discussion

This study confirmed that SBP can reduce myocardial I/R injury, improve myocardial reperfusion, and reduce myocardial infarction area, as well as improve cardiac function, in patients with STEMI.

Reperfusion therapy can reduce the area of myocardial infarction as well as the incidence of heart failure and mortality in patients with STEMI, which is the most effective way to treat STEMI (Bulluck et al., 2016). I/R injury is induced by reperfusion therapy, which usually occurs in STEMI patients Hausenloy and Yellon (2013), and accounts for more than 50% of myocardial infarction area. This reduces the effect of reperfusion therapy on STEMI patients (Hausenloy and Yellon, 2015; Bulluck et al., 2016). Myocardial infarction area is the main determinant of prognosis in patients with STEMI and is significantly related to cardiac function, cardiovascular adverse events, and cardiovascular death (Stone et al., 2016). Animal experimental studies have confirmed that many treatment measures can reduce myocardial I/R injury and myocardial infarction area (Hausenloy et al., 2017). However, there are few effective measures to reduce myocardial I/R injury and myocardial infarction area in STEMI patients, and clinical research results are inconsistently effective (Hausenloy and Bøtker, 2019). A meta-analysis involving nine randomized controlled trials (RCTs) and 1,220 patients found that remote ischemic conditioning (RIC) could reduce the myocardial infarction area (mean difference: 0.08; 95% confidence interval, 0.02–0.14) and increase the myocardial rescue index (mean difference: −2.46; 95% confidence interval, −4.66 to −0.26) (McLeod et al., 2017). Man et al. conducted a meta-analysis that included 13 RCTs and 1756 patients. The results indicated that RIC reduced the creatine kinase-myocardial band (CK-MB) (*p* = 0.0002), Troponin T (AUC, *p* = 0.003), and ST-segment resolution ≥70% (*p* = 0.03) (Man et al., 2017). However, in the recent CONDI-2/ERIC-PPCI study, 5401 STEMI patients were enrolled and RIC was not found to reduce myocardial infarction area (Troponin T AUC, *p* = 0.48) (Hausenloy et al., 2019). The METOCARD-CNIC study found that early injection of metoprolol reduced myocardial infarction area in patients with STEMI (25.6 ± 15.3 vs 32.0 ± 22.2, *p* = 0.012) (Ibanez et al., 2013). García-Ruiz et al. also found that early injection of metoprolol in patients with anterior STEMI could reduce myocardial infarction area (22.9 vs 28.1, *p* = 0.06) (García-Ruiz et al., 2016). However, a multicenter RCT study of 683 STEMI patients did not confirm that metoprolol injection could reduce myocardial infarction area (15.3 ± 11.0% vs 14.9 ± 11.5%, *p* = 0.616) (Li et al., 2019). Therefore, a single means treatment for myocardial I/R injury in STEMI patients may not be sufficient. Myocardial I/R injury has a multimodal pathogenesis and thus treatment of I/R injury may require targeting a variety of factors to achieve better results (Davidson et al., 2019; Li et al., 2019).

SBP is a traditional compound preparation composed of a variety of Chinese herbs Tian et al. (2018), diminishes the level of cardiac troponin T (cTNT) and cardiac troponin I (cTNI) in STEMI patients (Lu et al. (2018), significantly decreases the frequency of angina in patients with stable CAD Ge et al. (2021), and lower the MACCE in patients with NSTE-ACS (Zhou et al., 2016). However, we confirmed that SBP was able to reduce the myocardial infarction area and relieve I/R injury assessed by SPECT that accurately assessed I/R injury and myocardial infarction area (Bøtker et al., 2010). It was found that Muscone, a component of the SBP heart protecting pill, can reduce myocardial remodeling and improve heart function of STEMI rats by inhibiting the inflammatory response and apoptosis while upregulating hypoxia-induced vascular endothelial growth factor (Du et al., 2018a; Du et al., 2018b). However, no RCTs have reported that SBP reduces myocardial remodeling and improves heart function in STEMI patients. Our present study verified that SBP can improve heart function in STEMI patients due to its effect on reducing myocardial infarction area and relieving myocardial I/R injury. Myocardial I/R injury was decreased by inhibition of oxidative stress and apoptosis (Wu et al., 2011). Ginsenoside is the extract of Panax ginseng, which is a component of SBP; it can inhibit myocardial I/R injury and reduce myocardial infarction area by inhibiting oxidative stress, the inflammatory response, and apoptosis (Zheng et al., 2017; Li et al., 2018; Li et al., 2020). Cinnamaniline is an extract of cinnamon and a component of SBP, which also limits myocardial I/R injury in rats with AMI. The mechanism of action is to inhibit the opening of the mitochondrial permeability transition pore (MPTP), inhibit calcium overload and reduce oxidative stress (Fancelli et al., 2014). Therefore, the components of SBP can reduce myocardial I/R injury and myocardial infarction area by inhibiting the opening of MPTP, calcium overload, inflammatory response, oxidative stress, and anti-apoptosis (Wu et al., 2011; Fancelli et al., 2014; Zheng et al., 2017; Li et al., 2018; Li et al., 2020). Xiang et al. found that SBP can treat AMI rats by acting on inflammatory response, abnormal energy metabolism, abnormal amino acid metabolism, and oxidative damage (Xiang et al., 2012). SBP may participate in angiogenesis in MI rats throug up-regulation of 20-hydroxyeicosatetraenoic acid Huang et al. (2017) and activating macrophages to regulate endothelial cell function and signal transduction pathways (Zhang et al., 2020). Therefore, SBP may also have multiple mechanisms of action on myocardial I/R injury. The therapeutic effect of SBP on STEMI patients may be due to the synergistic action of multiple drugs on multiple myocardial I/R injury targets. The specific mechanism by which SBP acts is a main topic of future research. Meanwhile, Due to the defects of current pharmacological research methods, it is not enough to clearly demonstrate the mechanism of drugs with multiple targeted effects and molecular properties, which is also a challenge for this research at present.

In our study, SBP did not reduce MACCE (13.5 vs5.9%, *p* = 0.482). Possible reasons for this result are: 1) short follow-up time (Fancelli et al., 2014); and 2) the application of antiplatelet and statins may have diluted the curative effect of SBP(García-Ruiz et al., 2016). Our research also has some clinical limitations. The main reasons are too few samples, too short a follow-up time, and the fact that this was a single center study. A multicenter, randomized, double-blind, placebo-controlled clinical trial should be conducted to verify the therapeutic effect of SBP on I/R injury.

In conclusion, SBP can alleviate myocardial I/R injury, reduce myocardial infarction area, and improve cardiac function and myocardial reperfusion in patients with STEMI. If these results are confirmed in a large multicenter trial, SBP can become an effective measure against myocardial I/R injury, and may also become a part of the standard therapy for STEMI patients.

## Data Availability

The original contributions presented in the study are included in the article/Supplementary Material, further inquiries can be directed to the corresponding author.

## Abbreviations

AAR, area at risk of infarction; ACEI, angiotensin-converting enzyme inhibitor; ADAC, automatic direct analog computer; ARB, angiotensin receptor blockers; AMI, acute myocardial infarction; CHD, coronary heart disease; CK-MB, creatine kinase-myocardial band; cTNT, cardiac troponin T; cTNI, cardiac troponin I; ECG, electrocardiogram; FIS, final infarct size; hsTNT, high-sensitivity troponin T; I/R, ischemia/reperfusion; LVEF, left ventricular ejection fraction; MACCE, main adverse cardiovascular events; MBG, myocardial blush grade; MPTP, mitochondrial permeability transition pore; MS, myocardial salvage; NSTE-ACS, Non-ST-elevation acute coronary syndromes; NT-proBNP, N-terminal pro-B type natriuretic peptide; NT-proBNP, N-terminal pro-B type natriuretic peptide; PPCI, primary percutaneous coronary intervention; QPS, quantitative perfusion software; RCTs, randomized controlled trials; RIC, remote ischemic conditioning; SI, salvage index; STR, ST-segment resolution; SPECT, single-photon emission computed tomography; STEMI, ST-segment elevation myocardial infarction; TIMI, Thrombolysis in myocardial infarction.
